# Reporting a Rare Case of Pleomorphic Adenoma of the Breast

**DOI:** 10.1155/2014/387183

**Published:** 2014-05-13

**Authors:** Tahere Khamechian, Javad Alizargar, Tahere Mazoochi

**Affiliations:** ^1^Anatomical Sciences Research Center, Kashan University of Medical Sciences, Kashan, Iran; ^2^Student Research Committee, Kashan University of Medical Sciences, 5th Km-Qotb-e Ravandi Boulevard, P.O. Box 87155.111, Kashan 8199877475, Iran

## Abstract

Pleomorphic adenoma (PA) is the most common tumor type in the salivary gland. PA is uncommon in the breast tissue. Only 73 cases of PA of the breast have been reported in the world literature. We are reporting the 74th case of PA of the breast. A 61-year-old woman was referred to Shahid Beheshti Hospital Obstetric Clinic with bloody painless discharge from the right nipple. A bean size mass was detected immediately below the right nipple. After an excisional biopsy, the pathologist found proliferation in epithelial and myoepithelial cells that had small and multiple nuclei, myxoid and chondroid stroma. Immunohistochemistry stain was positive for S-100 and patchy for GFAP in tumor cells and for SMA around the tubule-glandular and tumor cell aggregates and suggested PA of the breast. It is essential for the pathologists to consider PA of the breast as a differential diagnosis of a rounded circumscribed mass in the juxta-areolar areas. Careful paraffin sections should be performed to avoid an unnecessary mastectomy.

## 1. Introduction

Pleomorphic adenoma (PA) or benign mixed tumor is the most common tumor type in the salivary gland [1]. Its uncommon sites are larynx, paranasal sinuses, palate, and nasal septum [2]. It also occurs in skin and is known as chondroid syringoma [1]. PA is uncommon in the breast tissue. The first case report was in 1906 when Lecène was the first one that reported PA in the breast [3]. From then only 73 cases of PA of breast have been reported in the world literature [4–7]. We are reporting the 74th case of PA of the breast reported to date.

## 2. Case Presentation

A 61-year-old woman with the history of hypertension and diabetes was referred to Shahid Beheshti Hospital Obstetric Clinic with bloody painless discharge from the right nipple. There was no nipple retraction. A bean size mass (maximum diameter of 2.2 cm) was detected immediately below the right nipple by palpitation. This mass was not adhered to the surrounding tissue. An excisional biopsy was taken from the mass. In the microscopic view, the pathologist found a neoplasm composed of two components: the epithelial component with the cells having round or ovoid nuclei and eosinophilic cytoplasm arranged in glandular or nest cell pattern and mesenchymal component containing myxoid and chondroid areas. The pathologist diagnosed the specimen as a benign mixed tumor. The specimen was sent to a second pathologist and also included immunohistochemistry (IHC) for a confirmation of the diagnosis. It was found that the neoplasm of the breast represented multiple rather circumscribed confluent nodules.

**Figure 1:**
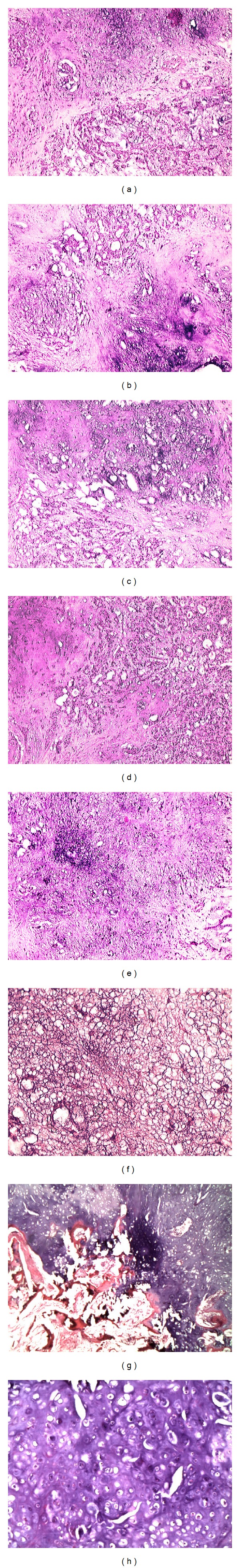
(a) and (b) Two components of PA, epithelial structure with glandular pattern and mesenchymal structure containing chondroid tissue and calcification. (c) Epithelial and mesenchymal components. (d) Predominance of epithelial component with glandular pattern. (e) Predominance of mesenchymal component with chondroid pattern. (f) Predominance of mesenchymal component with myxoid pattern. (g) Myxochondroid and osteoid pattern. (h) Mesenchymal component with predominance of chondroid tissue.

These nodules were composed of proliferated epithelial cells having round to slightly ovoid hyperchromatic nuclei and scant to moderate amounts of cytoplasm. They were arranged in nest aggregates and tubuloglandular structures and some cystic areas accompanied by mucocele formations. The stroma is mucohyaline (Figure 1). IHC stain was positive for S-100 and patchy for glial fibrillary acidic protein (GFAP-15) in tumor cells and for smooth muscle actin (SMA) around the tubule-glandular and tumor cell aggregates and negative for p53 which confirmed the diagnosis of the first pathologist. The second pathologist advised that this type of multinodular mixed tumor had more tendency to recur and should be removed with adequate excisional borders.

Within two weeks of the first diagnosis, excision of the mass was done and the surgery resulted in no residual tumor. There were no signs of recurrence after 1.5 years without any further adjunctive or radiotherapy.

## 3. Discussion

PA is a common type of tumor in salivary glands. Other sites for this kind of tumor have also been reported [2]. It has been 106 years since Lecène reported a case of PA in the breast [3]. From that date, 73 cases have been reported but rarely in Asian individuals. Differences in lactation habits between Asian and western women may be the reason [8]. Just 3 cases of PA in men have been reported [9–11]. Most tumors of PA occur in the right breast rather than left (right to left: 3/2). PA has the tendency to occur in the juxta-areolar region, and this may suggest that it originates from the large duct. Cases of breast PA occur in individuals of 23–85 years. Smith and Taylor in 1969 reported 9 cases of PA of the breast [12]. They believed that this kind of tumor is intraductal papillomas that has areas of osseous and chondroid stroma rather than a separate kind of neoplasm. Other authors believe that this kind of tumor is a separate entity. There is a possibility that the 9 cases were identical but not PA of the breast. But the presence of chondroid and osseous stroma, rare component of intraductal papillomas, makes us believe that the 9 cases were PA of the breast.

Minimum size of PA of the breast reported was 0.6 cm and the maximum size was 17 cm. The 17 cm tumor was of a patient that had this tumor for 30 years; but the majority of the tumors are reported to be 2 cm in size [4].

PA is usually characterized by epithelial or myoepithelial cells, myxoid and/or osseous matrix. Myoepithelial cell proliferation may be a key factor in tumorigenesis. Multipotency of ductal cells that differentiate into myoepithelial cells may be a key factor in this kind of tumor, suggested by Narita and Matsuda [8].

Adenocarcinoma with cartilage/osseous metaplasia, stromal sarcoma, phyllodes tumor, and fibroadenoma can be listed in differential diagnosis of PA of the breast. In 34 described cases, 33 cases had myxoid stroma and 27 had osteoid stroma [4]. Our case had both myoid and osteoid stromata.

This kind of tumor can be distinguished more easily by paraffin sections, but when a frozen section is the sole technique of diagnosing, the tumor may be misdiagnosed. This may be because of the lack of epithelial cells in every section that can be taken. It must be emphasized for a differential diagnosis with chondroid or osseous. In our study, the diagnosis was made upon a paraffin section. Epithelial component and myxochondroid and osteoid stroma convinced us to consider this kind of tumor as PA (Figure 1).

Therapeutic procedures when reviewing the literature were mostly excision, like our case, but three cases of PA of the breast have been reported with inevitable mastectomy [4]. One case that has been misdiagnosed as metaplastic carcinoma (osteoid-chondroid type) was overtreated with a mastectomy [7].

Local recurrence was reported in two cases [4, 13]. Multiple occurrences have been observed by Willen et al. [14], Moran et al. [15], and Sheth et al. [16]. Excision with clear margins is the preferred treatment for PA of the breast [4]. Most tumors of PA of the breast reported were benign. Just a few cases of carcinoma expleomorphic adenoma have been reported by Hayes et al. [17]. However, metastasis of PA has not been reported so far.

This original case report discusses the 74th case of PA of the breast, and it should be considered as a diagnosis when a patient presents with breast mass. It is essential for the pathologists to consider PA of the breast as a differential diagnosis of a rounded circumscribed mass in the juxta-areolar areas, and careful paraffin sections should be performed to eliminate an unnecessary mastectomy.
